# Investigation of the possible biological activities of a poisonous South African plant; *Hyaenanche globosa (Euphorbiaceae)*

**DOI:** 10.4103/0973-1296.59964

**Published:** 2010-02-13

**Authors:** Saeideh Momtaz, Namrita Lall, Ahmed Hussein, Seyed Nasser Ostad, Mohammad Abdollahi

**Affiliations:** 1 *Department of Plant Science, Faculty of Natural and Agricultural Science, University of Pretoria, Pretoria, South Africa*; 2 *Chemistry of Medicinal Plants Department, National Research Center, Dokki, Cairo, Egypt*; 3 *Faculty of Pharmacy, and Pharmaceutical Sciences Research Center, Tehran University of Medical Sciences, Tehran, Iran*

**Keywords:** *Hyenanche globosa*, hyenanchin, tutin, cytotoxicity, antibacterial assay, antioxidant assay, reactive oxygen species

## Abstract

The present study was undertaken to explore the possible biochemical activities of *Hyaenanche globosa* Lamb. and its compounds. Two different extracts (ethanol and dichloromethane) of four different parts (leaves, root, stem, and fruits) of *H. globosa* were evaluated for their possible antibacterial, antityrosinase, and anticancer (cytotoxicity) properties. Two pure compounds were isolated using column chromatographic techniques. Active extracts and pure compounds were investigated for their antioxidant effect on cultured ‘Hela cells’. Antioxidant/oxidative properties of the ethanolic extract of the fruits of *H. globosa* and purified compounds were investigated using reactive oxygen species (ROS), ferric-reducing antioxidant power (FRAP), and lipid peroxidation thiobarbituric acid reactive substance (TBARS) assays. The ethanolic extract of the leaves and fruits of *H. globosa* showed the best activity, exhibiting a minimum inhibitory concentration (MIC) of 3.1 mg/ ml and a minimum bactericidal concentration (MBC) of 1.56 and 6.2 mg/ml, respectively, against *M. smegmatis*. The ethanolic extract of the fruits of *H. globosa* (F.E) showed the highest percentage of inhibitory activity of monophenolase (90.4% at 200 μg/ml). In addition, F.E exhibited 50% inhibitory concentration (IC_50_) of 37.7 μg/ml on the viability of ‘HeLa cells’ using cytotoxicity MTT assay. Subsequently, F.E was fractionated using phase-partitioning with *n*-hexane, ethyl acetate, and *n*-butanol. The cytotoxicity of these fractions were determined *in vitro* using different cancer cell lines. The *n*-hexane fraction exhibited the highest activity of toxicity. Therefore, this fraction was subjected to further separation by chromatographic methods. Two pure compounds known as: ‘Tutin’ and ‘hyenanchin’ were isolated and their structures were determined by NMR spectroscopic methods. Unpredictably, none of them showed significant (*P* < 0.01) inhibition on cell viability/proliferation at the concentrations that were used. F.E showed significant anti-tyrosinase, antibacterial, and cytotoxicity effects, therefore it can be considered as an effective inhibitor alone or in combination with other plant extracts.

## INTRODUCTION

There is great scope for new drug discoveries based on traditional medicinal plant use throughout the world.[1] Nowadays, at least 25% of the active compounds in the currently prescribed synthetic drugs were first identified in plant resources[2] and 20000 plants have been used for medicinal proposes, of which, 4000 have been used commonly and 10% of those are commercial.

The Euphorbiaceae family is one of the largest families of plants, with about 300 genera and 7500 species of mostly monoecious herbs, shrubs, and trees that are further frequently characterized by a milky sap or latex material. Members of Euphorbeaceae family have been investigated for providing potential treatments for cancer, tumors, and warts.[3] The chemistry of Euphorbiaceae is one of the most diverse and interesting one of the flowering plant families and is comparable to the biological diversity of the family. Of all chemical classes, the most useful for a chemotaxonomic study of the Euphorbiaceae, above the level of genus, appear to be alkaloids, cyanogenic glycosides, diterpenes, glucosinolates, tannins and triterpenes. *Hyaenanche globosa*, Lamb. (Euphorbeaceae) is a narrow endemic plant and is restricted to a single flat-topped mountain near Van Rhynsdrop in southern Namaqualand. This plant is the single species of *Hyaenanche*. It is a small, rounded tree, with dark green, leathery leaves, characteristically arranged in four along the stems. Male and female flowers are both small and occur on separate trees. The fruits are large rounded capsules with several segments. *Hyaenanche* is a Greek word for hyena poison and was chosen because the fruits were formerly used to poison carcasses in order to destroy hyenas and other vermin. This plant contains several toxic sesquiterpene lactones, such as, tutin, mellitoxin, urushiol III, and isodihydrohyaenanchine. Its main toxin, tutin, is known to cause convulsions, delirium, and coma in humans.[1–2] In the present study our aim was to examine the possible bioactivities of *H. globosa*.

## MATERIALS AND METHODS

### Chemicals and reagents

Fetal bovine serum RPMI 1640, and penicillin/streptomycin were obtained from Gibco, Inc. (UK). MTT (3-(4,5-dimethylthiazol-2-yl)-2,5-diphenyl-tetrazolium bromide) powder, DCF-DA (2,7-dichlorofluorescin diacetate), 2,4,6-tripyridyl-s-triazine (TPTZ), and all the other chemicals and reagents were obtained from Sigma-Aldrich (UK). FeCl_3_.6H_2_O, sodium sulfate, and FeSO_4_, 2-thiobarbituric acid (TBA) were obtained from Merck (Germany). *L*-Tyrosine, *L*-DOPA, tyrosinase, arbutin and kojic acid were obtained from Sigma-Aldrich (Kempton Park, South Africa). All chemicals and solvents were of the highest commercial grade.

### Preparation of plant extracts

The *H. globosa* (leaves, roots, stem, and fruits) materials were collected from the Botanical Garden of the University of Pretoria during May 2007. The plant was identified at the H.G.W.J. Schwelckerdt Herbarium (PRU) of the University of Pretoria (Voucher herbarium specimen number: S.M. 95499). Forty grams of each powdered part (shade dried) was soaked in 200 ml of ethanol and dichloromethane separately for four hours and after filtration the solvents were removed under vacuum (BUCHI, Rotavapor, R-200) to yield dry extracts (F.E: Fruits, ethanol extract; F.DC: Fruits, dichloromethane extract; L.E: Leaves, ethanol extract; F.DC: Leaves, dichloromethane extract; R.E: Root, ethanol extract; R.DC: Root, dichloromethane extract; S.E: Stem, ethanol extract; S.DC: Stem, dichloromethane extract).

### Antibacterial bioassay against mycobacterium smegmatis

The minimum inhibitory concentration (MIC) and minimum bactericidal concentration (MBC) of the extracts were determined as described previously.[4–5] The sample extracts were dissolved in 10% dimethyl sulfoxide (DMSO) in a sterile Middlebrook 7H9 broth base, to obtain a stock concentration of 50.0 mg/ml. Serial two-fold dilutions of each sample to be evaluated were made with 7H11 broth, to yield volumes of 200 μl/wells, with final concentrations ranging from 12.5 mg/ml to 0.390 mg/ml. The highest percentage of DMSO (10%), which was not toxic to bacteria, was used in this assay. Ciprofloxacin at a final concentration of 0.156 mg/ml, served as a positive drug control.

### Inhibition of tyrosinase activity and DOPA auto-oxidation

This assay was performed using methods as described earlier.[6–7] The extracts were dissolved in DMSO to a final concentration of 20 mg/ml. This extract stock solution was then diluted to 600 μg/ml in a 50 mM potassium phosphate buffer (pH 6.5). The extracts were tested only at two concentrations, 20 and 200 μg/ml, for their inhibitory effect on the monophenolase and diphenolase activated forms of tyrosinase *in vitro*. Arbutin and kojic acid (positive controls) were also tested at the above-mentioned concentrations. In a 96-well plate, 70 μl of each extract dilution was combined with 30 μl of tyrosinase (333 units ml in phosphate buffer) in triplicate. After incubation at room temperature for 5 minutes, 110 μl of substrate (2 mM *L*-tyrosine or 12 mM *L*-DOPA) was added to each well. Incubation commenced for 30 minutes at room temperature. The optical densities of the wells were then determined at 492 nm with the BIOTEK PowerWave XS multi-well plate reader (A.D.P., Weltevreden Park, South Africa).

### Isolation of active constituents

**Figure 1:**
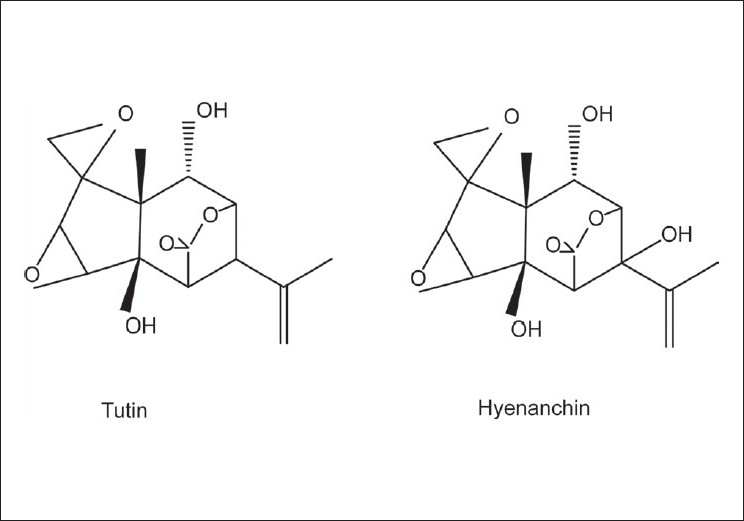
Chemical structures of the isolated compounds from the ethanolic extract of F. E (fruits, ethanol extract) of *H. globosa*

The ethanolic extract of the fruits of *H. globosa* (F.E) exhibited the highest cytotoxicity effect of ‘Hela cells’ compared to the other extracts. The ethanolic extract was selected for the isolation and identification of active principle(s). One thousand two hundred grams of air-dried fruits of the plant were milled into a fine powder using a commercial grinder. The powder was extracted thrice, each time with 3 L of ethanol at 50°C for 24 hours. The combined ethanol extract was filtered and the filtrate was concentrated to dryness under reduced pressure in a rotary evaporator. The dried ethanolic extract of the fruits of *H. globosa* (70 g) was redissolved in 80% ethanol (ethanol/distilled water; 75:25) and partitioned with *n*-hexane and ethyl acetate. The organic layers were evaporated to dryness at 40°C to give 22 g, 28 g, and 18 g of *n*-hexane, ethyl acetate, and aqueous fractions, respectively. The bioassay of these fractions of *H. globosa* showed that the *n*-hexane fraction demonstrated the highest inhibition of cell growth/proliferation (82% at 100 μg/ml) in the ‘Hela cells’. It was therefore subjected to fractionation on a Silica gel column LH-20 (7 × 50 cm) using a gradient of *n*-hexane:ethyl acetate of increasing polarity (0 to 100% ethyl acetate). Forty-two fractions were collected and those with similar thin-layer chromatography (TLC) profiles were combined. TLC plates were developed using (*n*-hexane: ethyl acetate; 9:1) as eluent. Acidic vanillin was used as a detecting agent. Fractions exhibiting similar TLC profiles were combined together to provide 14 major fractions (1B to 14B). The pure compound ‘tutin’ was crystallized from 12B spontaneously (white hairy crystals, yield: 456 mg; 0.038%) [Figure 1]. Fractions 13B and 14B (2.645 g) were chromatographed separately using silica gel column LH-20 (Sigma-Aldrich, South Africa) using *n*-hexane:ethyl acetate of increasing polarity (0 to 90% ethyl acetate) as an eluent, to obtain pure ‘hyenanchin’ from 13B (white rounded crystals, yield: 347 mg; 0.028%) [Figure 1]. The compounds were identified by mass spectrometric and NMR data, which were identical to those in the literature.

### Cell culture

Six cancerous cell lines HT29/219 (Human, Colon, epithelial-like, Carcinoma), HeLa (Human, Cervix, epithelial-like, Carcinoma), Caco_2_ (Human, Colon, Adenocarcinoma), NIH-3T3 (Swiss NIH mouse, embryo fibroblast), K562 (Human, Pleural effusion, Lymphoblast-like) and T47D (Human, Breast, ductal-carcinoma), and one normal cell line (HPLF) were purchased from the Pasteur Institute, Tehran, Iran. The cells were maintained in RPMI 1640, supplemented with 10% fetal bovine serum, 0.28 units/ml insulin, 100 μg/ml streptomycin, 100 units/ml penicillin, and 0.3 mg/ml glutamine. The cells were grown at 37°C in a humidified atmosphere of 5% CO_2_, in air.

### Cytotoxicity

The cytotoxicity of the different extracts of *H. globosa* and the isolated compounds from the ethanolic extract of fruits (tutin and hyenanchin) was assayed using the MTT cytotoxicity assay.[8] The cells (3 × 10^4^) were plated in 500 μl of medium/well in 48-well plates (NUNC Cell Culture Flasks, Denmark). After an overnight incubation at 37°C, in 5% CO_2_, and a humidified atmosphere, the extracted samples were added to the cells to a final concentration of 500 μg/ ml. ‘Metotherexate’ (positive control) and pure compounds were examined at concentrations ranging from 5, 10, 20, 40, 80, and 100 μg/ml. The plates were incubated at 37°C, in 5% CO_2_, humidified atmosphere, for 48 hours. After 48 hours, 50 μl of 5 mg/ml MTT (dissolved in PBS) was added per well. After three hours of incubation, the MTT solution was removed and the cells were washed with 100 μl of PBS, twice. One hundred and fifty microlitres of DMSO was added per well, to solubilize the formazan crystals. The optical densities of the wells were then measured at 570 nm (690 nm reference wavelength). By referring to the control (medium with DMSO), the cell survival was assessed.

### Preparation of cells for ferric-reducing antioxidant power and lipid peroxidation thiobarbituric acid reactive substance assays

As mentioned earlier (section 2.7), ‘HeLa cells’ (1X10^6^) were seeded in 25-cm^2^ cell culture flasks (Falcon) (NUNC Cell Culture Flasks, Denmark) in a minimum essential medium RPMI 1460 (Gibco, UK), until nearly confluent. After an overnight incubation at 37°C, in 5% CO_2,_ and a humidified atmosphere, F.E was added to the cells to form final concentrations of 12.5, 25, 50, 100, 200, and 400 μg/ml. Tutin and hyenanchin (isolated pure compounds) were examined at concentrations ranging from 5, 10, 20, 40, 80, and 100 μg/ml. The plates were incubated at 37°C, in 5% CO_2_, and a humidified atmosphere for 48 hours. Thereafter, the medium was removed and 2 ml of ‘trypisin’ was added to each flask to harvest the cells. The cells were centrifuged at RPM 2000 for five minutes and were resuspended in PBS, twice. The pellets were used for FRAP and TBARS.

### Ferric-reducing antioxidant power assay

Following the procedures as described by Dehghan *et al.*,[9] the total antioxidant capacities of (F.E), tutin, and hyenanchin were determined by measuring the ability of the medium to reduce Fe^3+^ to Fe^2+^. The complex between Fe^2+^ and TPTZ gave a blue color, with absorbance at 593 nm.

### Thiobarbituric acid reactive substance assay

Assay of TBARS is the method of choice for screening and monitoring lipid peroxidation, a major indicator of oxidative stress. To precipitate the cell's proteins, 500 μl of TCA 20% (m/V) was added into 100 μl ml of the sample, which was then centrifuged at 1500 × g for 10 minutes. Then 500 μl of sulfuric acid (0.05 ml^−1^) and 400 μl TBA (0.2%) were added to the sediment, shaken and incubated for 30 minutes in a boiling water bath. Subsequently, 800 μl *n*-butanol was added and the solution was centrifuged, cooled, and the supernatant absorption was recorded at 532 nm, using a Synergy4 BIOTEK multi-well plate reader (BIOTECK, USA). The calibration curve was obtained using different concentrations of 1, 1, 3, 3-tetramethoxypropane as a standard to determine the concentration of TBA-MDA adducts in samples.[10–11]

### Measurement of intracellular reactive oxygen species

This assay was performed using methods as described by Yong Sun and Wang,[12–13] with slight modifications. On day one, 1 × 10^4^ number of ‘HeLa cells’ were seeded in 96-well black fluorescent cell culture plates. The intracellular generation of ROS was measured using the oxidation-sensitive fluorescent dye 2,7-dichlorofluorescin diacetate (DCF-DA). On the second day, the cells were incubated with 500 μl of different concentrations of samples (12.5 to 400 μg/ ml for the sample extract (F.E) and 3.1 to 100 μg/ml for pure compounds. After an hour the medium was removed and the cells were washed with HBSS (Life Technologies, Inc.) twice. The cells were then incubated with 500 μl of HBSS containing 10 μg/ml of DCF-DA for 15 minutes at 37°C. The fluorescence intensity of dichlorofluorescein was measured at 530 nm emission wavelength, after excitation at 480 nm, at 10-minute intervals, for up to 90 minutes using a Synergy4 BIOTEK multi-well plate reader (BIOTEK, USA). An increase in fluorescence intensity was used to represent the generation of net intracellular ROS. Nontreated cells were used as negative control in contrast to H_2_O_2_ as positive control in concentrations of 125 to 2000 mM.

## RESULTS AND DISCUSSION

Nowadays, the discovery of novel phyto-pharmaceuticals from natural sources is extremely encouraging. Despite the variety and frequency of the Euphorbiaceae species, very little information on the medicinal values of *H. globosa* is available. Based on the available information about the toxicity effect of the fruits of this species,[2] it is being considered that the other parts also might have the same effects. To explore the possible bioactivities of *H. globosa*, two different extracts (dichloromethane and ethanol) of fruits, leaves, roots, and stem were prepared separately. The antibacterial, anticancer, and anti-tyrosinase activities of these extracts were examined.

Of the eight different extracts of *H. globosa*, in antimicrobial assay, R.DC and L.DC were found to be the most effective. They exhibited MIC values of 0.39 mg/ml against *M. smegmatis*. The L.E and F.E were the next best extracts, which inhibited growth at 3.13 mg/ml. The F.DC, R.E, S.DC, and S.E had the same MIC of 6.25 mg/ ml. Ciprofloxacin (positive drug control for *M. smegmatis*) inhibited the growth of bacteria at a concentration of 0.15 mg/ml [Table 1]. Mativandlela[7] reported that the ethanolic extracts of *Artemisia afra*, *Drosera capensis*, and *Galenia africana* exhibited MIC values of 1.56, 3.1, and 0.78 mg/ml against *Mycobacterium smegmatis*. Comparison of the data obtained in this study with the previously published results shows that the antibacterial activities of the different extracts of *H. globosa* are promising, even better than some pure compounds. Epigallocatechin, catechin, and umckalin, isolated from the butanolic extract of the root of *Pelargonium sidoides* showed a minimum inhibitory concentration (MIC) of 7.8, 31.25, and 62.5 mg/ ml, respectively against *M. smegmatis*.[6]

**Table 1:** Antibacterial activity of different extracts of *H. globosa* against *M. smegmatis*

Samples	MIC^a^ (mg/ml)	MBC^b^ (mg/ml)
F.E	3.1 ± 0.4	6.2 ± 1.4
F.DC	6.2 ± 0.9	3.1 ± 0.6
L.E	3.1 ± 0.6	1.5 ± 0.6
L.DC	0.39 ± 0.4	25 ± 3.3
R.E	6.2 ± 1.1	1.5 ± 2.7
R.DC	0.39 ± 0.7	25 ± 3.4
S.E	6.2 ± 1.3	6.2 ± 0.4
S.DC	6.2 ± 4.1	NA^c^
CIP	0.15 ± 0.1	3.12 ± 1.8

F.E - Fruits, ethanol extract; F.DC - Fruits, dichloromethane extract; L.E - Leaves, ethanol extract; F.DC - Leaves, dichloromethane extract; R.E - Root, ethanol extract; R.DC - Root, dichloromethane extract; S.E - Stem, ethanol extract; S.DC - Stem, dichloromethane extract; CIP - Ciprofloxacin;

a Minimum inhibitory concentration

b minimum bactericidal concentration

c NA, no activity at highest concentration tested; Data are mean ± SD of three separate experiments

The ethanolic extracts from the fruits, leaves, and roots of *H. globosa* showed 90.4, 87, and 86.8% inhibition of tyrosinase activity at 200 μg/ml (*P* < 0.01), respectively. They also demonstrated 31, 8.4, and 13.7% inhibition of DOPA auto-oxidation, respectively, at 200 μg/ml (*P* < 0.01). Other extracts showed a marginal inhibition of tyrosinase and DOPA auto-oxidation activity. Kojic acid significantly showed 100% inhibition of monophenolase activity at 200 μg/ml (*P* < 0.01), while arbutin exhibited 32.4% anti-tyrosinase activity (*P* < 0.01). The inhibition of *L*-DOPA auto-oxidation was determined as 83.3 and 0% by Kojic acid and arbutin, respectively [Table 2]. In our previous study, the methanolic extract of the leaves of *H. globosa* showed 92 and 42% inhibition of monophenolase and diphenolase activities at 500 μg/ml, respectively.[14] Another publication reviewed, *Glycyrrhiza glabra*, *Morus alba*, and *Gastrodia ellata* (80% ethanol extract), which showed 65, 68, and 85% tyrosinase inhibition at the concentration of 333 μg/ml, respectively.[15]

**Table 2:** Inhibitory activities of mushroom tyrosinase and DOPA auto-oxidation by different extracts of *H. globosa*

Sample	% Inhibition of DOPA auto-oxidation (%) at 20 μg/ml	Inhibition of DOPA auto-oxidation (%) at 200 μg/ml	Inhibition of tyrosinase (%) at 20 μg/ml	Inhibition of tyrosinase (%) at 200 μg/ml
F.E	15.7 ± 0.03	31.7 ± 0.05	13 ± 0.01	90.4 ± 0.03
F.DC	15.5 ± 0.02	19.1 ± 0.09	0	1.8 ± 0.02
L.E	0	8.4 ± 0.06	4.8 ± 0.04	87 ± 0.02
L.DC	13.3 ± 0.03	13.6 ± 0.02	0	0
R.E	9 ± 0.03	13.7 ± 0.02	53.8 ± 0.03	86.8 ± 0.06
R.DC	14.8 ± 0.06	18.3 ± 0.02	0	0
S.E	0.9 ± 0.03	0	0	40.2 ± 0.01
S.DC	7.4 ± 0.04	10.1 ± 3	0	0
Kojic acid	42.2 ± 0.2	83.3 ± 0.2	99 ± 0.1	100 ± 0.5
Arbutin	0	0	8.7 ± 0.8	32.6 ± 0.1

F.E - Fruits, ethanol extract; F.DC - Fruits, dichloromethane extract; L. E - Leaves, ethanol extract; F.DC - Leaves, dichloromethane extract; R. E - Root, ethanol extract;R. DC - Root, dichloromethane extract; S.E - Stem, ethanol extract; S.DC - Stem, dichloromethane extract Data are mean ± SD of three separate experiments

In recent years, the anti-cancer property of various Sesquiterpene lactones has attracted a great deal of interest and extensive research has been carried out to characterize the anticancer activity, the molecular mechanisms, and the potential chemotherapeutic application of them.[16] Among the eight different extracts of *H. globosa*, R.E and F.E demonstrated IC_50_ values of 46.5 μg/ml and 37.7 μg/ ml of the ‘HeLa cells,’ respectively (*P* < 0.01). The other extracts did not show significant inhibition of the cell growth or proliferation [Table 3]. Accordingly, the F.E phase partitioned into three fractions (section 2.5), of which the n-hexane fraction demonstrated the highest inhibition of ‘HeLa cell’ growth/proliferation (82% at 100 μg/ml). Subsequently, two known pure compounds; tutin and hyenanchin were isolated. Following this, their cytotoxicity and antioxidant activities were examined using conventional methods. F.E exhibited IC_50_ values of 25.1, 25.9, 82.1, >120, >120, and 49.2 μg/ml when Caco2, Hela, HT29, NIH3T3, K562, and T47D were used, respectively. Fuentealba[17] reported the concentration-dependent inhibitory effect of tutin, obtained from *Coriaria ruscifolia* subspecie ruscifolia, on spinal glycine receptors. In another study, tutin isolated from the essential oils of the Pimpinella species, was not cytotoxic to the mammalian cells that were explored (SK-MEL, SK-OV3, BT-549, and KB).[18] The literature review showed an epileptogenic action by tutin, derived from Coriaria Lactone, (a mixture that has been used to establish animal models of epilepsy) in rats, demonstrating that tutin is a potent convulsant.[19] Hall[20] found that tutin and hyenanchin were present in common foods, such as potatoes, rice, carrots, and honey. Their safety depended on the amount of tutin and hyenanchin present in the food. The bioactivities that are reported in this study are novel, and to the best of our knowledge there are no other multi-sides about *H. globosa* that have been studied to date.

**Table 3:** Effect of eight different extracts of *H. globosa* on the viability of ‘HeLa cells’ using MTT assay

Samples	IC_50_ (μg/ml)
F.E	37.7 ± 3.2
F.DC	>120
L.E	>120
L.DC	>120
R.E	46.5 ± 4.6
R.DC	>120
S.E	>120
S.DC	>120

F.E - Fruits, ethanol extract; F.DC - Fruits, dichloromethane extract; L.E - Leaves, ethanol extract; F.DC - Leaves, dichloromethane extract; R.E - Root, ethanol extract; R.DC - Root, dichloromethane extract; S.E - Stem, ethanol extract; S.DC - Stem, dichloromethane extract Data are mean ± SD of three separate experiments

Tutin and hyenanchin did not show any significant reduction on cell viability/proliferation on the tested cell lines. The IC_50_ value of > 60 μg/ml was observed for all samples in the ‘HPLF’ normal control cells [Table 4]. The effect of Methotrexate (anticancer drug) on the viability of different cancer cell lines has been shown in Table 5.

**Table 4:** Effect of *H. globosa* on the viability of different cancer cell linesby using MTT assay

Cell lines	F. E IC_50_ (μg/ml)	Tutin IC_50_ (μg/ml)	Hyenanchin IC50 (μg/ml)
HeLa	25.9 ± 0.8	>120	>120
NIH3T3	>120	>120	>120
T47D	61.5 ± 0.1	>120	>120
Caco2	25.1 ± 0.02	>120	>120
HT29	82.1 ± 0.3	>120	>120
K562	>120	>120	>120
HPLF	>60	>60	>60

F.E - Fruits, ethanol extract Data are mean ± SD of three separate experiments

**Table 5:** Effect of Methotrexate on the viability of different cancer cell lines using MTT assay

Cell lines	Methotrexate IC_50_ (μg/ml)	Methotrexate IC_50_ (nmol)
L929	0.46 ± 0.03	101.2
NIH3T3	0.24 ± 0.013	50
T47D	0.16 ± 0.09	31
Caco2	0.23 ± 0.04	70.4
HT29	0.23 ± 0.02	50

Methotrexate as an anticancer drug was used as a positive control. Data are mean ± SD of three separate experiments

Only few investigations have been performed that led to the isolation of a few active principles of this plant. As mentioned before, *H. globosa* contains several toxic sesquiterpenes, such as, tutin, mellitoxin, urushiol III, and isodihydrohyaenanchine.[1–2,21] Several studies have reported that tutin is the major neurotoxin in the New Zealand shrubs of the genus Coriaria. Kinoshita[22] succeeded in isolating tutin from the acetone extracts of achenes separated from the *Coriaria japonica* berries. The hydroxy derivative hyenanchin (also called mellitoxin) is a major active component in toxic honey.[23–25]

Various compounds derived from the plant's secondary metabolites are commonly used in cancer chemotherapy, but only a few are potent and effective. The MTT analysis showed that pristimerin (triterpinoid), isolated from *Maytenus ilicifolia Martius* (ethanolic extract of root bark) exhibited IC_50_ values of 0.55 to 3.2 μg/ml, against MDA/MB435 and K-562.[26]

Sayyah[27] described that the essential oil of the leaves of *Croton flavens* exhibited IC_50_ values of 27 ± 4 μg/ml for A-549 (human lung carcinoma) and 28 ± 3 μg/ml for DLD-1(human colon adenocarcinoma). In another study,[28] two fractions of *Myrica gale* (60-minute and 30-minute fractions) were assessed against A-549 and DLD-1. The 60-minute fraction showed higher anticancer activity against both tumor cell lines with an IC_50_ value of 88 ± 1 μg/ml. The 30-minute fraction had an IC_50_ value of 184 ± 4 μg/ml for A-549 and 160 ± 3 μg/ml for DLD-1. The higher cell growth inhibition induced by the 60-minute fraction, as compared to the 30-minute fraction, could be due to sesquiterpene enrichment.

**Figure 2:**
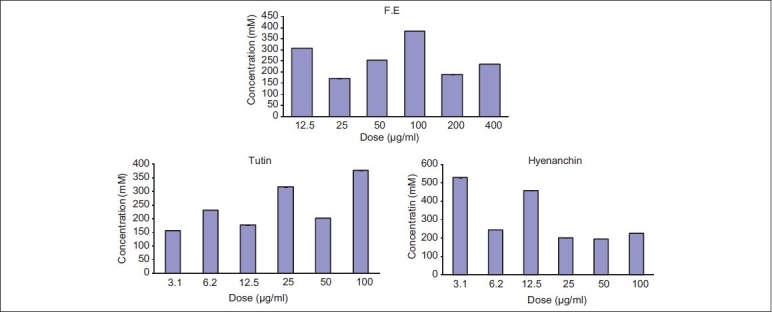
Ferric-reducing antioxidant power potential of F. E (fruits, ethanol extract), tutin, and hyenanchin in cultured ‘HeLa cells’

**Figure 3:**
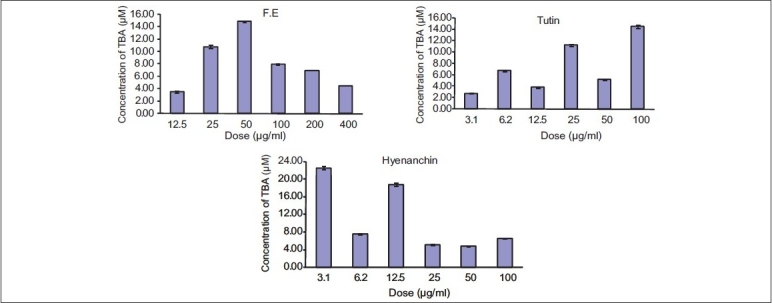
Lipid peroxidation TBARS potential of the F. E (fruits, ethanol extract), tutin, and hyenanchin TBARS in cultured ‘HeLa cells’

The mean TBARS in the control cells were 399.6 μmol/L, reaching 170.7 μmol/L, in treated ‘HeLa cells’ by F.E (*P* < 0.01) [Figure 2]. Treatment of cells with pure compounds could not decrease the cell TBARS, significantly (*P* < 0.01, Figure 3).

**Figure 4:**
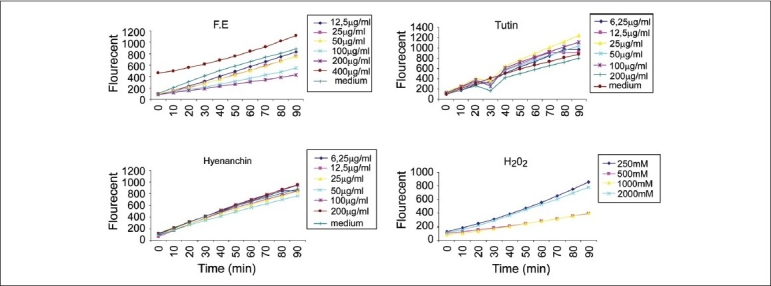
Time-response curve of increase of DCF fluorescence in ‘HeLa cells’ after 90 minutes exposure to various concentrations of F. E (fruits, ethanol extract), tutin, and hyenanchin. Each data point represents the mean of data from three wells (n = 3)

As a standard for an ROS assay (to compare the production of ROS), we first tested H_2_O_2_ to explore the concentration-response relationship of the exposed cells. Figure 4 shows that the levels of ROS detected with the fluorescent dye DCFDA in the ‘HeLa cells’ demonstrated an enhancement with time in all the samples. Among pure compounds, the ROS level did not seem to jump up very much further than the control, but F.E exhibited a very good level of ROS production at 400 μg/ml.

## CONCLUSION

In summary, in spite of our great expectation about the toxicity of pure compounds isolated from the ethanolic extract of the fruits of *H. globosa* (tutin and hyenanchin), they did not show any significant cytotoxic effects on the examined cancer cell lines, while the crude extract was well known for its poisonous effects. The poisonous effect of this plant could be due to the activity of the compounds that were not isolated yet. It could be concluded that the ethanolic extract of the fruits of *H. globosa* showed significant anti-tyrosinase, antibacterial, and cytotoxic effects, therefore, it could be considered as an effective inhibitor alone or in combination with the other plant extracts. Although the data are still inconclusive and further scientific attempts are needed to confirm the traditional information or to investigate the novel medicinal aspects of this plant. A further study aims to determine the anticancer properties of other major constituents of *H. globosa*, as well as identify the unknown compounds required to fully understand its bioactivity.
